# Antibacterial Activity and Biocompatibility of Ag-Montmorillonite/Chitosan Colloidal Dressing in a Skin Infection Rat Model: An In Vitro and In Vivo Study

**DOI:** 10.3390/jfb14090470

**Published:** 2023-09-12

**Authors:** Kaining Yang, Lei Shen, Lin Zhang, Wenxin Sun, Yuhong Zou, Yande Ren, Rongchang Zeng

**Affiliations:** 1 Department of Bioengineering, College of Chemical and Biological Engineering, Shandong University of Science and Technology, Qingdao 266590, China; conyoung@sdust.edu.cn (K.Y.); leilmy2022@163.com (L.S.); lenny4772@163.com (W.S.); 2 Hospital of Shandong University of Science and Technology, Qingdao 266590, China; moonzhanglin@163.com; 3 Affiliated Hospital of Medical College Qingdao University, Qingdao 266555, China; 4 College of Materials Science and Engineering, Shandong University of Science and Technology, Qingdao 266590, China

**Keywords:** silver-loaded montmorillonite, chitosan, colloidal dressing, infection model, rat, antimicrobial properties

## Abstract

(1) Background: Traditional dressings can only superficially cover the wound, they have widespread issues with inadequate bacterial isolation and liquid absorption, and it is simple to inflict secondary wound injury when changing dressings. Therefore, it is crucial for wound healing to develop a new kind of antimicrobial colloidal dressing with good antibacterial, hygroscopic, and biocompatible qualities. (2) Methods: Ag-montmorillonite/chitosan (Ag-MMT/CS) colloid, a new type of antibacterial material, was prepared from two eco-friendly materials—namely, montmorillonite and chitosan—as auxiliary materials, wherein these materials were mixed with the natural metal Ag, which is an antibacterial agent. The optimum preparation technology was explored, and Ag-MMT/CS was characterized. Next, Staphylococcus aureus, which is a common skin infection bacterium, was considered as the experimental strain, and the in vitro antibacterial activity and cytocompatibility of the Ag-MMT/CS colloid were investigated through various experiments. Subsequently, a rat skin infection model was established to explore the in vivo antibacterial effect. (3) Results: In vitro studies revealed that the Ag-MMT/CS colloid had a good antibacterial effect on *S. aureus*, with an inhibition zone diameter of 18 mm and an antibacterial rate of 99.18%. After co-culture with cells for 24 h and 72 h, the cell survival rates were 88% and 94%, respectively. The cells showed normal growth and proliferation, and no evident dead cells were observed under the laser confocal microscope. After applying the colloid to the rat skin infection model, the Ag-MMT/CS treatment group exhibited faster wound healing and better local exudation and absorption in the wound than the control group, suggesting that the Ag-MMT/CS colloid exhibited a better antibacterial effect on the *S. aureus*. (4) Conclusions: Ag^+^, chitosan, and MMT present in the Ag-MMT/CS colloid dressing exert synergistic effects, and it has good antibacterial effects, cytocompatibility, and hygroscopicity, indicating that this colloid has the potential to become a next-generation clinical antibacterial dressing.

## 1. Introduction

Dressing has been widely used in wound protection and repair as it can establish a relatively enclosed and sterile space, absorb the wound ooze fluids, and promote tissue regeneration. Traditional dressings such as gauze, bandages, skimmed cotton, and non-woven fabrics can only simply cover the wound surface, which universally has weak germ isolation ability and poor fluid absorption ability. In particular, when changing dressings, it can easily cause secondary damage to the wound and increase the patient’s pain and wound healing difficulty. There were some attempts to introduce new materials as dressing materials. For example, O. Akturk et al. [1] prepared a new dressing using collagen/gold nanoparticle composite material, which can inhibit the inflammatory response and promote wound closure effectively. Others have tried to improve the structure of traditional dressings. For example, Cheng et al. [2] prepared a composite gauze with directional fluid absorption ability by using a hydrophobic polypropylene nonwoven layer as the contact layer and cotton gauze as the absorbent layer, which solved the problem of contact adhesion between traditional gauze and wound peripheral tissues and water retention by gauze dressing leading to compression of skin tissue at the wound site affecting wound healing, etc. Modern medical demands have pushed the development of dressings continuously in the direction of being more efficient and multifunctional; thus, the development of new antibacterial, hygroscopic, and other multifunctional dressings has become an inevitable trend.

Bacterial infection is the key factor and significant obstacle to wound healing. Infection prolongs the inflammatory response, hinders re-epithelialization and collagen synthesis, and delays the healing process [3]. Dressings with antimicrobial properties can act directly on the wound surface, which has unique advantages in reducing drug toxicity and medication costs compared to systemic administration. The antibacterial ability of the material can be improved by using certain metal ions [4]; the current ones with good application are Ag^+^, Cu^2+^, and Zn^2+^ [5,6,7]. Among them, Ag^+^ is strong in bactericidal ability and low in cytotoxicity; it is the most widely used metal ion antimicrobial substance at present [8]. Chitosan (CS) belongs to a natural organic antimicrobial agent with good broad-spectrum antimicrobial properties. It inhibits biofilm formation and promotes wound healing and tissue growth [9,10]. It is often used as an antimicrobial agent and drug delivery vehicle due to its good biodegradability and biocompatibility [11,12,13]. Currently, various antimicrobial dressings and drug carriers using chitosan are approved by the US Food and Drug Administration (FDA) [14].

Carrier materials are also important for the effectiveness of antimicrobial properties of applications. In recent years, inorganic non-metallic materials have become a new choice for antimicrobial agent carriers, such as natural minerals containing Si. Montmorillonite (MMT), a natural silicate with a lamellar structure, is often used as a carrier for inorganic and organic antimicrobial materials due to its outstanding cation exchange capacity and adsorption [15], e.g., Our previous works [16,17] have prepared Zn-MMT and GS-MMT antibacterial materials, and found that both materials had significant inhibitory effects on *E. coli* and *S. aureus*. In this project, Ag-MMT was prepared by using MMT as the carrier material and Ag^+^ as the antibacterial element; it was then dispersed in a chitosan solution to prepare Ag-MMT/CS colloid [18]. As a novel biomaterial, its biosafety is critical. Currently, a variety of cytotoxicity testing tools, including the MTT method, can accurately and rapidly determine whether a biomaterial is potentially biohazardous or contains toxic substances [19]. Therefore, along with the in vivo and in vitro characterization and study of the antimicrobial properties of Ag-MMT/CS colloids, we also designed cytotoxicity tests specifically with MC3T3-E1 cells to ensure the biosafety of the material.

We hypothesize that this novel wound dressing has good antibacterial and biocompatible properties and hygroscopicity, which is important for reducing the side effects of antibiotics and wound healing.

## 2. Materials and Methods

### 2.1. Ethical Approval

All animal experiments were approved by the medical ethics committee of the Affiliated Hospital of Medical College Qingdao University (approval date: 20 June 2023; approval number: QYFY WZLL 26750) and performed according to all applicable international, national, and institutional guidelines for the care and use of animals.

### 2.2. Experimental Materials

The carrier used for the experiments was Na-MMT (pharmaceutical grade, Zhejiang Fenghong Ltd. (Hangzhou, China). LP0042 tryptone, LP0021 yeast extract, and CM0367 agar were purchased from Thermo Fisher Scientific Ltd. (Waltham, MA, USA). Chitosan (100 kDa, ≥95% deacetylation) was purchased from Shanghai Macklin Biochemical Co., Ltd. (Shanghai, China). Silver nitrate, sodium chloride, sodium hydroxide, etc., were analytical pure reagents provided by Tianjin Tiantai Fine Chemicals Ltd. (Tianjin, China). and Yantai Shuang Shuang Chemical Ltd. (Yantai, China). Wistar rats were purchased from Qingdao Qinda Biotechnology Ltd. (Qingdao, China). MC3T3-E1 cells were purchased from iCell Bioscience Inc. (Shanghai, China). *S. aureus* strains were self-stored and activated in the laboratory.

### 2.3. Preparation of Materials

#### 2.3.1. Single-Factor and Orthogonal Experiments

To investigate the effect of each factor on the amount of silver loaded in MMT, single-factor experiments were conducted on the four influencing factors of AgNO_3_ concentration (A), time (B), temperature (C), and pH (D). Then, four-factor three-level orthogonal experiments were designed on this basis to finally obtain the optimal preparation conditions for Ag-MMT. The table of four-factor three-level orthogonal experiments is shown in Table 1.

#### 2.3.2. Preparation of Ag-MMT/CS Colloid

**Figure 1:**
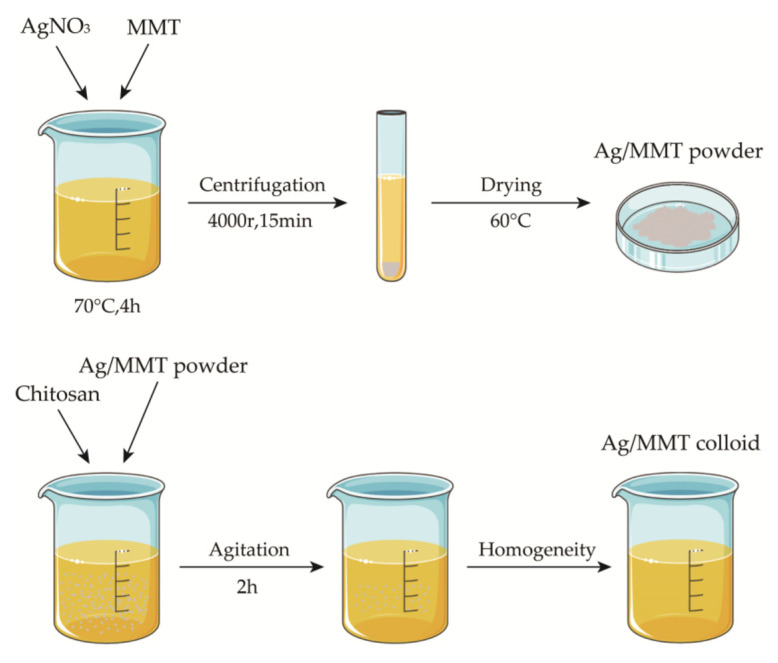
Schematic diagram for fabrication process of Ag-MMT/CS colloid.

Weigh 5 g of sodium montmorillonite into 100 mL of deionized water, stir for 5 h at room temperature to obtain the suspension, weigh a certain amount of AgNO_3_ into the suspension, adjust the pH, heat, and stir for 4 h at a constant temperature, then centrifuge for 15 min at 4000 r. Discard the supernatant, wash the precipitate repeatedly, dry to a constant weight, grind, and pass through a 200 mesh sieve to obtain silver-loaded montmorillonite (Ag/MMT). Then, weigh 1.5 g of chitosan into 50 mL of 1% glacial acetic and stir for 5 h at room temperature until uniform to obtain 3% (*w*/*w*) chitosan colloid. Then, add 1.5 g of Ag/MMT powder and stir for 2 h at 50 °C to obtain Ag-MMT/CS colloid. We used the SNB-2 viscometer to measure the viscosity of Ag-MMT/CS colloid, CS colloid, and deionized water at room temperature (25 °C). The preparation process is shown in Figure 1.

### 2.4. Characterization of the Materials

The material morphology was observed by scanning electron microscopy (Nova Nano SEM 450, Hillsboro, OR, USA), and the chemical bonding states of Ag-MMT and Ag-MMT/CS were identified by FTIR spectroscopy (NicoletiS 50, Thermo Fisher Scientific Ltd., Waltham, MA, USA). The crystal structures of the samples were analyzed by X-ray diffractometer (D/MAX-2500PC, Rigaku, Tokyo, Japan).

### 2.5. Cytotoxicity Assay

Cytotoxicity assay was conducted using MC3T3-E1 cells in the logarithmic phase, and the cell suspension was adjusted to a seeding density of 1 × 10^4^ cells/well. The material was sterilized and placed into a 6-well culture plate. MC3T3-E1 cells were seeded into the plate and cultured at 37 °C and 5% CO_2_. After culturing for 24 h, the cells were washed with PBS solution. Next, 4 mL of medium containing 0.5 mg·mL^−1^ MTT was added to the culture plate. The medium was then removed after culturing for 4 h. Dimethyl sulfoxide (DMSO; 1.5 mL) was then added to each well, and the plate was placed on a shaker at low speed for 10 min to dissolve the crystal completely. MMT was used as the control, the cell culture medium was used as the negative control, and medium containing 10% DMSO was used as the positive control. The absorbance of each well was measured at 570 nm using an enzyme-linked immunosorbent assay.

MC3T3-E1 cells were cultured at a seeding density of 4 × 10^4^ cells/well and incubated with the material for 24 h. Then, a mixture of the material and the medium was extracted and washed with PBS solution. Calcein and AM were added, after which the plate was incubated at 4 °C for 15 min, followed by washing with PBS solution and staining with propidium iodide for 5 min. Photographs were captured using a confocal microscope. Viable cells were indicated in green, and dead cells were indicated in red.

### 2.6. Antibacterial Activity

*S. aureus* were picked from the slant of the test tube and inoculated in the corresponding liquid medium, after which the medium was cultured overnight on a shaker at 37 °C for 12 h. The thalli were collected by centrifugation and washed twice with phosphate-buffered saline (PBS) solution (50 mmol/L, pH 6.5). The buffer solution was then added to prepare a bacterial suspension for subsequent use.

#### 2.6.1. Inhibitory Activity

The concentration of the bacterial solution was diluted to 10^7^ CFU/mL for spectrophotometry analysis, and 0.1 mL of the diluted bacterial suspension was uniformly coated on the petri dish.

A clean filter paper was cut into circular disks of 10 mm diameter each, after which the disks were immersed into Ag-MMT/CS colloid, montmorillonite, chitosan, and silver nitrate (AgNO_3_) to prepare the respective filter paper disks. These filter paper disks were placed on culture-containing petri dishes and then incubated for 18–24 h in an incubator at 37 °C. The inhibition zones were then measured.

#### 2.6.2. Bacterial Growth Curve

The *S. aureus* bacterial solution that was in the logarithmic growth phase was inoculated into the liquid culture broth medium at a 1% inoculum size (thalli concentration: 10^8^ bacterial cells/mL). Ag-MMT/CS colloid, montmorillonite, chitosan, and AgNO_3_ were added into the respective culture medium and then cultured on a shaker at 37 °C and 150 rpm. Samples were assessed at regular intervals; after culturing, the samples were measured using a spectrophotometer at OD_650_ (*S. aureus*) to construct the growth curve of *S. aureus*.

#### 2.6.3. Plate Colony Counting

The *S. aureus* strains were inoculated on the slant with inoculation loops at room temperature for 20 min on the ultra-clean bench and incubated in a constant temperature incubator at 37 °C for 16–18 h. The slants were rinsed with saline to obtain bacterial suspensions, which were diluted to OD_650_ = 0.1 by visible spectrophotometry to obtain dilutions, and further diluted 10^−4^ times with Ag-MMT/CS colloid and sterile water. Take 0.1 mL of the bacterial suspension dilution and spread it evenly on the solid plate, incubate it in the incubator at 37 °C for 16–18 h, and then count the plate. After counting, the inhibition rate of the sample was calculated according to the inhibition rate Formula (1). Inhibition formula:

Inhibition rate (%) = (control group colonies − experimental group colonies)/control group colonies × 100%.(1)

#### 2.6.4. Analysis of the Rat Infection Model

**Figure 2:**
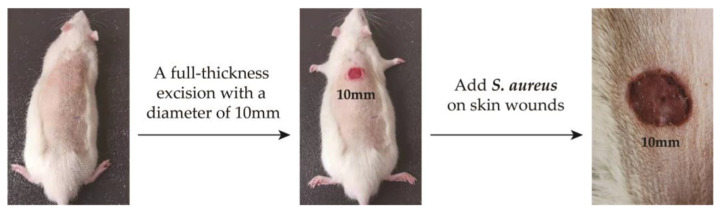
Establishment of skin wound infection model in rats.

The experimental animals were Wistar rats purchased from Shandong Provincial Laboratory Animal Center. All animal experiments were conducted according to the ISO 10993-2:1992 animal welfare requirements. The protocol for their care and the use of laboratory animals was approved by the Animal Ethical Committee of the Affiliated Hospital of Qingdao University. All animal experiments were in accordance with the MMT/CS colloid treatment group and control group (n = 5, each group). All the Wistar rats were weighed and anesthetized with pentobarbital sodium (40 mg kg^−1^) by intraperitoneal injection. And then, we made a 10mm diameter hole on the back of the rat. Then, 100 μL of *S. aureus* solution with OD_650_ = 0.1 was injected into the wounds of the rats, and the changes in body temperature and skin wounds were observed. After 4 days, the two groups of rats developed fever and the wound showed obvious redness and swelling. The infection model was successfully established (labeled as D4, Figure 2).

When the infection model was established, the treatment group started to receive Ag-MMT/CS colloid via taking 0.2 mL Ag-MMT/CS colloid and applying it evenly on the wound surface once a day for 10 days. At the same time, the survival status, body temperature, and wound healing of the rats continued to be observed. The wounds of the two groups of rats were photographed on days D4, D7, and D14. Tissues were taken locally on D1, D4, and D14 days, fixed in 10% paraformaldehyde, sectioned, stained, and analyzed pathologically.

### 2.7. Ag^+^ Release Determination

Ag-MMT/CS colloid dressings were submerged in phosphate buffered saline (PBS) at 37 °C for 24 h to investigate the Ag^+^ release behavior. Then, 1 mL of PBS solution was removed at a regular interval and 1 mL of freshly PBS solution was added. Concentration of Ag^+^ released from Ag-MMT/CS colloid was measured using ICP-MS (inductively coupled plasma mass spectrometry). Triplicates were performed for each measurement to yield accumulative release curves of Ag^+^.

### 2.8. Statistical Analysis

The data obtained were analyzed using Jade 6.5, Origin 2023b, Photoshop 2023, and SPSS 26.0, and the mean X ± standard deviation (SD) was used to record the experimental results. A *t*-test was performed on the experimental data results; *p* < 0.05 indicated significant differences, and *p* < 0.01 indicated highly significant differences.

## 3. Results

### 3.1. Single-Factor Experiment

**Figure 3:**
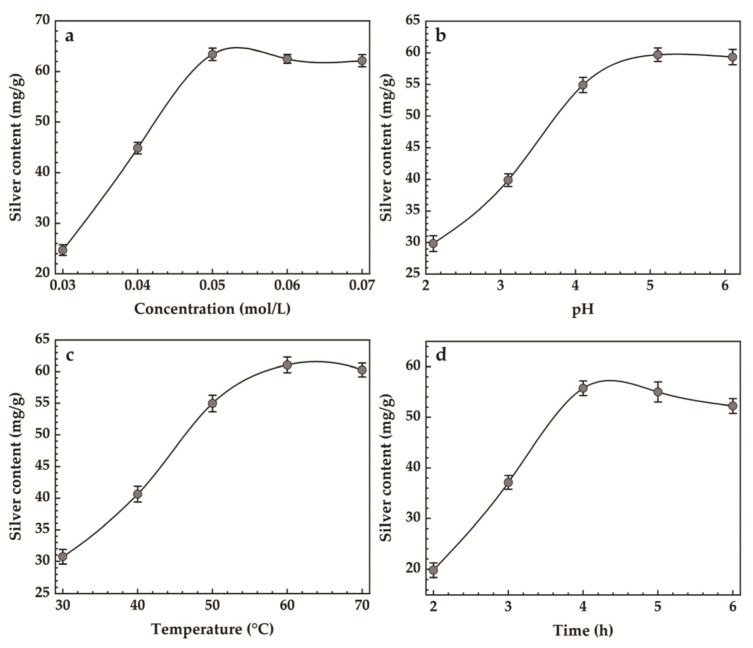
Effect of (**a**) concentration, (**b**) pH, (**c**) temperature, (**d**) time on silver content.

The results of single-factor experiments for Ag-MMT preparation are shown in Figure 3. It can be seen that the maximum silver content was achieved when the AgNO_3_ concentration was 0.05 mol/L from Figure 3a. Figure 3b shows that the maximum silver loading was achieved when the pH reached 5.4. In Figure 3c, it can be seen that the silver content increased gradually with the increase in heating temperature, and when the temperature reached 60 °C, the maximum silver content was 61.07 mg/g, and then it decreased slightly. Figure 3d shows that when the heating time reaches 4 h, the maximum amount of silver content reaches 55.53 mg/g.

### 3.2. Orthogonal Experiments

The results of the intuitive analysis are shown in Table 2. The “mean values 1, 2, and 3” in the table indicate the mean average of the silver loading capacity of montmorillonite at the level of each factor, which can determine the optimal level that should be obtained for each factor. The extreme difference R of the mean adsorption capacity at the level of the same factor reflects the magnitude of the effect of the change in the level of each factor on the experimental results. Among the factors influencing the silver loading of montmorillonite, AgNO_3_ concentration (A) had the most significant effect, followed by heating time (B) and pH (D), and temperature (C) had the least effect. The process conditions with the highest silver loading were as follows: A2B2C3D1 at four factors, i.e., AgNO_3_ 0.05 mol/L, heating time 4 h, temperature 70 °C, and pH 4.4.

### 3.3. Material Characterization

**Figure 4:**
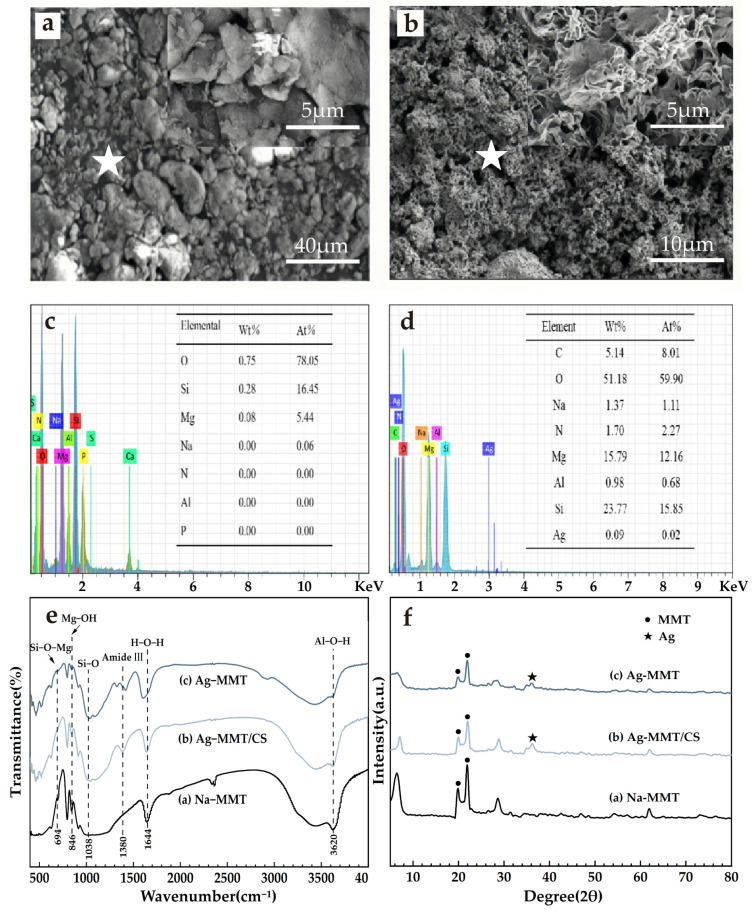
SEM surface morphology (**a**) Na-MMT, (**b**) Ag-MMT; corresponding EDS plots (the star is the location of selected EDS scan point) and elemental fraction of (**c**) Na-MMT and (**d**) Ag-MMT; XRD patterns of (**e**) Ag-MMT, Ag-MMT/CS and Na-MMT; FTIR spectra for (**f**) Ag-MMT, Ag-MMT/CS and Na-MMT.

The results of material characterization are shown in Figure 4. It can be seen from Figure 4a that the Na-MMT particles are larger and aggregated into flake-packed agglomerates with rounded shield, swirled, and sharp-angled edges under electron microscopy. Figure 4b shows that Ag-MMT particles are finer and irregularly shaped, and some edges also have lamellar swirling morphology. Under high magnification, it can be seen that the sample has a typical lamellar structure of montmorillonite. EDS are shown in Figure 4c,d. In combination with Figure 4c,d, the presence of Ag^+^ in montmorillonite is confirmed by the presence of Ag elements in the Ag-MMT material.

XRD and FT-IR results are shown in Figure 4e,f. In Figure 4e, it shows that the characteristic peaks belonging to MMT appear at diffraction angles 2θ of 19.78 and 21.78 with no significant difference, and the diffraction peak of Ag appears at diffraction angle 2θ of 36.3, indicating the Ag^+^ agglomeration between the layers. XRD indicates that Ag^+^ is successfully intercalated into montmorillonite. Figure 4f shows that the main characteristic peaks of montmorillonite appear in the composite at 694 cm^−1^, 846 cm^−1^, and 1038 cm^−1^, corresponding to Si-O-Mg, the bending vibration of Mg-OH, and the stretching vibration of Si-O, respectively. The characteristic peak of chitosan appears at 1380 cm^−1^, which corresponds to the stretching vibration of the amide III band. All of the above indicate that the basic structure of montmorillonite was not changed during the preparation of the composites, and its original properties were retained.

### 3.4. Cytotoxicity Test

**Figure 5:**
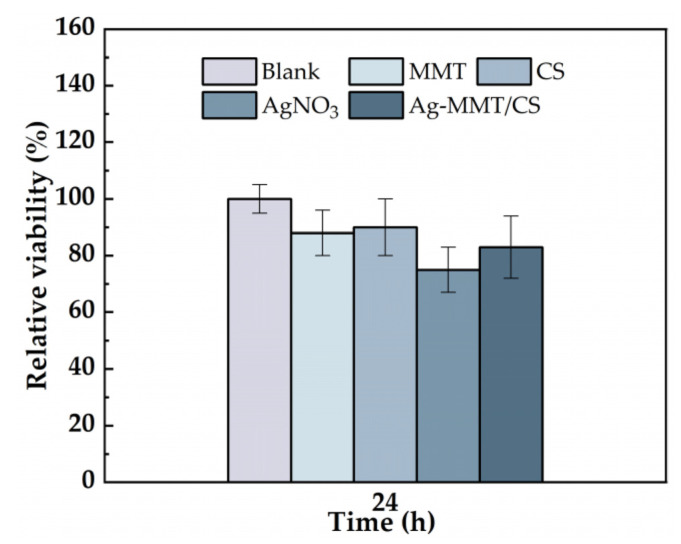
Cell viability incubated for 24 h with blank, MMT, CS, AgNO_3_, and Ag-MMT/CS (*p* ≥ 0.05).

The cytotoxicity of the Ag-MMT/CS colloid were determined using an MTT assay, and shown in Figure 5. After 24 h of co-culture of materials and cells, the cell survival rates in MMT, chitosan, AgNO_3_, and Ag-MMT/CS colloid were 87%, 90%, 75%, and 88%, respectively. Statistical analysis shown that pairwise comparison has no significant (*p* ≥ 0.05). This result shows that Ag-MMT/CS colloid, MMT, and chitosan had good cytocompatibility, as the components are eco-friendly materials. Silver ions in AgNO_3_ have certain toxicity to cells, but owing to their low dosage in the Ag-MMT/CS colloid, they do not affect the cytocompatibility.

### 3.5. Live/Dead Cell Staining Experiment

**Figure 6:**
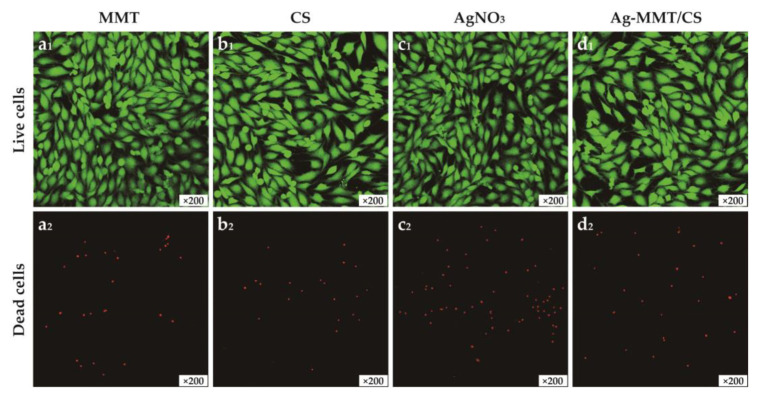
The images of live/dead staining of MC3T3-E1 cells after 24 h culturing on MMT(**a_1_**,**a_2_**), CS(**b_1_**,**b_2_**), AgNO_3_(**c_1_**,**c_2_**), and Ag-MMT/CS(**d_1_**,**d_2_**).

Figure 6 shows the images of MMT, chitosan, AgNO_3_, and Ag-MMT/CS colloid cultured with cells for 24 h. The growth of cells treated with the four materials remained normal, and no evident dead cells were seen, which is consistent with the cytotoxicity assay results. This result shows that the Ag-MMT/CS colloid exhibits good cytocompatibility.

### 3.6. Antibacterial Properties of Ag-MMT/CS

#### 3.6.1. Inhibition Zone Test

**Figure 7:**
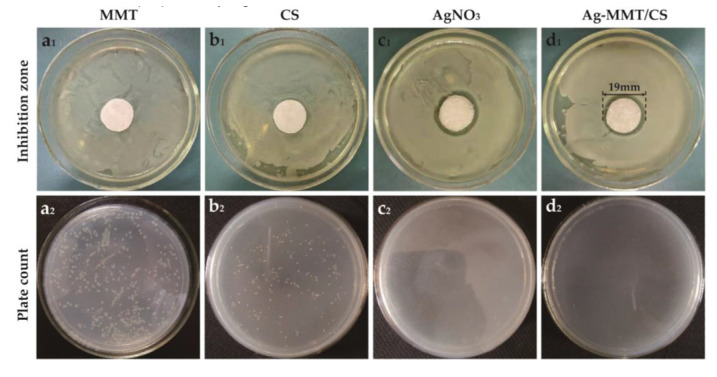
Inhibition zone (**a_1_**–**d_1_**) and plate count (**a_2_**–**d_2_**) of MMT, CS, AgNO_3_, and Ag-MMT/CS against *S. aureus*.

Figure 7 demonstrates the inhibition zones around the Ag-MMT/CS-colloid-containing filter paper disks on *S. aureus* after culturing at a constant temperature for 24 h. No inhibition zone was found around the MMT (Figure 7a_1_), whereas inhibition zones were observed around filter paper disks containing chitosan, AgNO_3_, and Ag-MMT/CS colloid (Figure 7b_1_–d_1_) with diameters of 8 mm, 17 mm, and 19 mm, respectively. This finding suggests that all three materials have antibacterial effects on *S. aureus*; however, the inhibition zone of the Ag-MMT/CS colloid was the largest, and the antibacterial effect of the colloid was better than those of AgNO_3_ and chitosan.

#### 3.6.2. Colony Counting Assay

Figure 7a_2_–d_2_ shows the zone of inhibition and plate count results for MMT, CS, AgNO_3_, and Ag-MMT/CS. After 16–18 h, the number of colonies of *S. aureus* in CS, AgNO_3_, and Ag-MMT/CS colloid decreased by 22.7%, 99.7%, and 99%, respectively. The calculated antibacterial rate of the Ag-MMT/CS colloid was 99.18%. This finding showed that the Ag-MMT/CS colloid has a strong antibacterial effect on *S. aureus*.

#### 3.6.3. Bacterial Growth Curve

**Figure 8:**
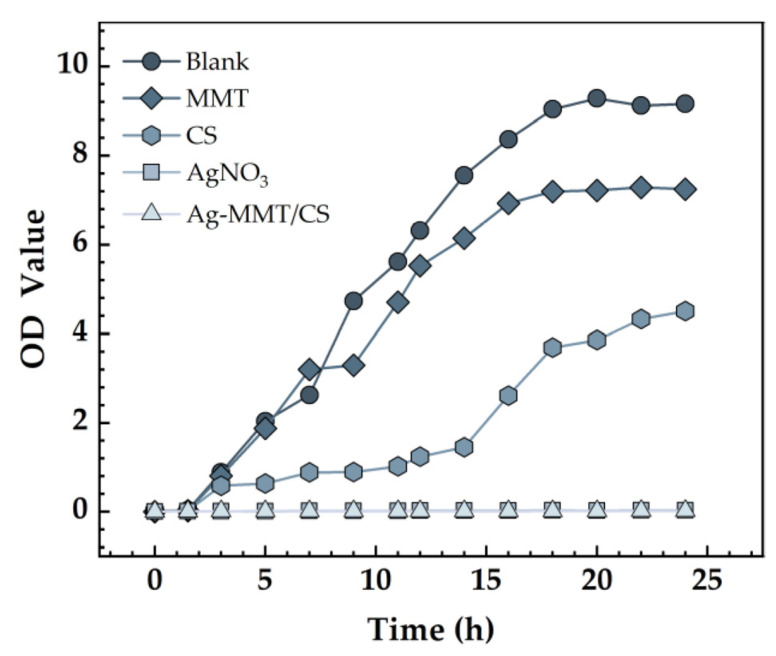
Growth curves *S. aureus*.

The effect of Ag-MMT/CS colloid on the growth curve of *S. aureus* is shown in Figure 8. The growth curve of *S. aureus* in the blank control and MMT groups was S-shaped, which is the typical bacterial growth curve, indicating that MMT coating has no inhibitory effect on *S. aureus*. The absorbance value of the bacterial solution in the Ag-MMT/CS colloid and AgNO_3_ groups showed no significant difference, indicating that the growth of *S. aureus* was significantly inhibited. This result indicates that the Ag-MMT/CS colloid has a good antibacterial effect on *S. aureus*.

#### 3.6.4. Analysis of the Rat Infection Model

**Figure 9:**
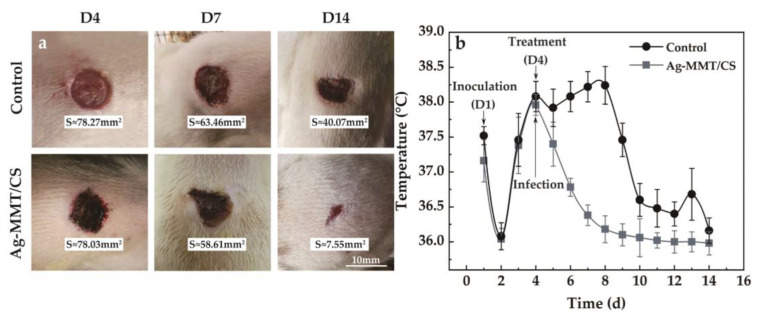
(**a**) Wound healing after treatment; (**b**) the body temperature of the rat model of infection.

The viscosity of Ag-MMT/CS colloid, CS colloid, and deionized water are 3487 mPa·s, 1882 mPa·s, and 2 mPa·s, respectively. The larger viscosity of Ag-MMT/CS colloid is conducive to its adhesion to skin wounds. The treatment of Ag-MMT/CS colloid on the rat skin infection model is shown in Figure 9a. The thickness of the colloidal dressing applied to the wound surface gradually decreases and eventually forms a light film. It can be seen from D14 that the wound color of the colloid treatment group is black, which is the color of Ag in Ag-MMT/CS.

After 10 days, the wound size of the control group and the colloid-treated group was 40.07 mm^2^ and 7.55 mm^2^—they decreased to the original size of 51.19% and 9.68%, respectively. The wound healed significantly in the colloid-treated group, while the wound area in the untreated control group did not change much. The colloid-treated group had faster wound healing and better wound healing ability compared with the control group.

The changes in the body temperature of rats during the infection treatment are shown in Figure 9b. It can be seen that the body temperature of rats started to change on the fourth day after Ag-MMT/CS colloid treatment, and the average body temperature of rats in the dressing treatment group reached the highest point of 37.9 ± 0.3 °C. Then, the body temperature started to decrease, and all of them dropped to below 37 °C on the sixth day. In the control group, the body temperature reached 38.5 ± 0.6 °C on the eighth day, and then gradually decreased to normal. This indicates that Ag-MMT/CS colloid has a better antibacterial effect on skin infection in rats that can be caused by *S. aureus*; it can shorten the fever time in rats and play a certain role in promoting wound healing.

#### 3.6.5. Rat Wound Pathology

**Figure 10:**
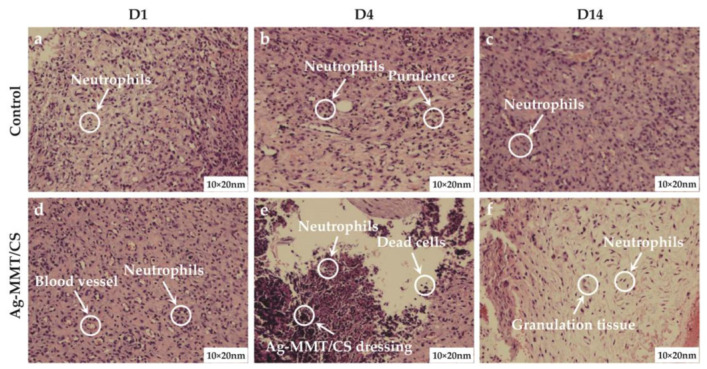
H and E staining of skin wound on the D1, D4, and D14.

The results of skin wound pathology are shown in Figure 10. As shown in Figure 10a,d, after skin perforation (D1), a small number of neutrophils were present in the wound tissue in both groups, indicating a mild inflammatory reaction. Figure 10b,e shows that a large number of neutrophil infiltration and local purulent cells appeared in pathological sections of both the treatment group and the control group on the 4th day of wound establishment (D4). Figure 10c,f shows that after 10 days of colloid treatment (D14), a large amount of granulation tissue and lymphocytes appeared in the wound tissue of the rats in the Ag-MMT/CS colloid treatment group, and neutrophils were significantly reduced, indicating that the wound basically started to heal. The control group still had obvious neutrophil infiltration. This indicates that the Ag-MMT/CS colloid has good antibacterial properties and can inhibit the infection of germs in the wound.

### 3.7. Ag^+^ Release Determination

**Figure 11:**
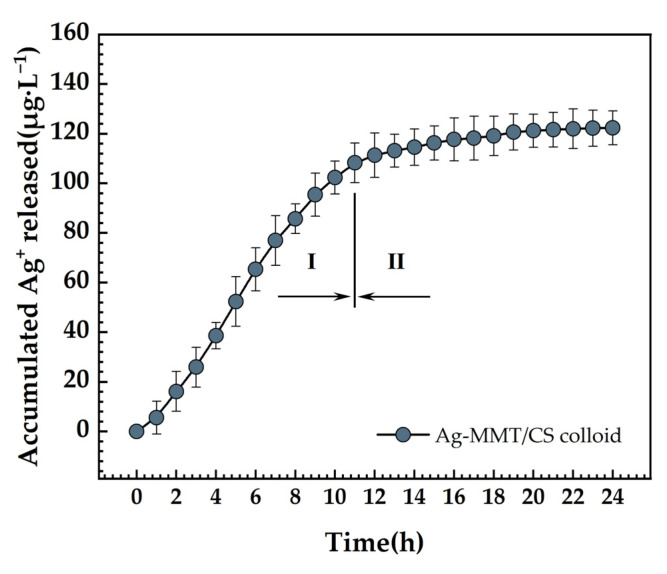
Ag^+^ release curve of Ag-MMT/CS colloid in PBS for 24 h.

The accumulative release curve of the Ag^+^ is presented in Figure 11. There are essentially two steps to the release of Ag^+^. The release rate is higher in the initial stage and reaches 108.12 μg·L^−1^ at 11 h. After 11 h, it moved into the second stage, where the rate of Ag^+^ release was relatively modest, and the total amount released was 120.33 μg·L^−1^ after 24 h. First, the Ag^+^ attached to the MMT surface was rapidly released, which became the primary Ag^+^ release mode. Then, the Ag^+^ of the MMT lamellae began to detach slowly. Finally, the long-lasting release of Ag^+^ was realized.

## 4. Discussion

Modern wound dressings are under more pressure than ever before due to expanding and shifting medical needs. They should not only provide basic wound covering but also have specific water absorption, antimicrobial, and mechanical stability qualities [20]. Today, researchers’ optimization and improvement objectives are rapidly shifting towards multi-functionality rather than being restricted to a single attribute.

This topic follows the trend of new medical dressing development. The Ag-MMT intermediate was created using the ion exchange method to intercalate Ag^+^ into the Na-MMT lamellae by cation exchange. The release rate of Ag^+^ from Ag-MMT/CS colloid displayed a trend similar to other researcher on the MMT-sustained release drugs with a fast-slow trend. In the early stage, the rapid release of Ag^+^ came from the rapid dissolution of physically adsorbed Ag^+^ on the surface of MMT. Ag^+^ between the layers of MMT were released slowly, which lasted for 24 h, as we mentioned in “3.7 Ag^+^ release determination”. Then, the Ag^+^ works on bacterial cells to change the permeability of the cell membrane, obstruct DNA replication, and stop cell division. Ag^+^ also interferes with the synthesis of peptidoglycan by RNA, preventing the formation of the cell wall [21]; it also binds to the enzyme molecules that synthesize ATP in the bacterial cell wall, inhibiting the formation of ATP necessary for the metabolic activity of the bacterial cell [22] and rendering the bacterial cell inactive. It has also been suggested that the antibacterial activity of silver is due to the interaction of silver and sulfhydryl groups in bacterial proteins [23].

The Ag-MMT is mixed with chitosan, which then electrostatically adsorbs to the Ag-MMT surface to create the Ag-MMT/CS colloid. It is worth noting that MMT acts primarily as a carrier for Ag^+^ and does not have an antimicrobial effect. CS has two main roles: First, it provides adhesion to immobilize the dressing to the wound surface. Second, CS is a natural antibacterial agent and has a good antibacterial effect on *S. aureus.* It has two main antimicrobial mechanisms: one is that the positively charged amino groups on the polymer chains of chitosan interact electrostatically with the negatively charged components of microbial membranes, resulting in antimicrobial properties [24]. Another possible mechanism is that chitosan acts as a chelating agent that causes damage to the integrity of microbial membranes by sequestering iron, zinc, copper, cadmium, magnesium, and other divalent cations, leading to toxin production and inhibition of microbial growth [25].

The Ag^+^ chosen for material preparation has broad-spectrum antimicrobial properties. It has no drug resistance compared with traditional antibiotics. In addition, due to the unique lamellar structure of MMT, Ag^+^ can realize slow release, that is, it can persistently play an antimicrobial effect. Chitosan plays a positive role in preventing tissue adhesion and reducing scarring of wounds and it can also create a hydrophilic film on the skin, preventing water loss [26]. It has also been reported that plasma and extracellular matrix proteins are involved in enhancing platelet adhesion when chitosan is present, which has a significant beneficial effect on wound healing [27]. Thus, the colloidal dressing made of chitosan can closely adhere to the skin surface, providing a moist, non-adherent, and favorable environment for wounds.

The Ag-MMT/CS colloid we prepared has good antibacterial properties, and the results of the circle of inhibition experiments show that it can produce a 19 mm diameter circle of inhibition against *S. aureus*. Many traditional antibiotic materials based on MMT can also produce effective antimicrobial effects against *E. coli*. Parolo et al. [28] reported that the circles of inhibition of composites of Tetracycline and Minocycline with Na-MMT as the carrier were 19.0 mm and 17.2 mm, respectively. Rapacz-Kmita et al. [29] indicated that the circles of inhibition of composites of Gentamicin and Neomycin based on Poly-l-lactide/MMT were 8 mm (rough surface), 19 mm (smooth surface), 7.6 mm (rough surface), and 15.3 mm (smooth surface), respectively. Therefore, among MMT-based antimicrobial materials, the Ag-MMT/CS colloidal dressing has the better bacteriostatic ability.

MMT is an ideal slow-release drug carrier, which is not absorbed by the body and does not enter the blood circulation. Its crystal surface and loose interlayer structure can attract water molecules, so it has a good hygroscopic ability, which can absorb local exudate and reduce wound exudation effectively. MMT can form charged interactions with clotting factors so that clotting factors are effectively activated, and thus also has certain coagulation functions [30]. In addition, it has also been shown that MMT can adsorb negatively charged microorganisms and significantly increase the antimicrobial activity of the material [31].

Safety is a constant theme in biomaterial research. The primary requirement for medical materials that are in direct contact with the human body is that they do not have a large toxic effect on the organism. Although the cytotoxicity of the three basic materials, Ag^+^, CS, and MMT, varies at different concentrations [32,33,34], we have demonstrated the safety of the dressings prepared with these three basic materials at the concentrations we set, which provides a favorable prerequisite for further in vitro and clinical testing of the dressings.

## 5. Conclusions

A multifunctional Ag-MMT/CS colloid dressing with good antibacterial, cytocompatibility, and hygroscopicity was successfully prepared in this study. The following conclusions were drawn:

(1) The Ag-MMT/CS colloid showed a typical montmorillonite lamellar structure under the electron microscope. EDS showed the presence of Ag. XRD showed characteristic peaks attributed to MMT appearing at 19.78° and 21.78°, consistent with FT-IR.
(2) The Ag-MMT/CS colloid showed a good in vitro antibacterial effect on *S. aureus*, and the inhibition zone had a diameter of 19 mm. The bacterial growth curve showed that the colloid could significantly inhibit *S. aureus*, with the antibacterial rate reaching 99.18%.
(3) The Ag-MMT/CS colloid demonstrated good cytocompatibility, indicating that it is an eco-friendly dressing.
(4) The Ag-MMT/CS colloid has the advantages of fast wound healing and good hygroscopicity in a rat skin wound infection model.

Ag^+^, chitosan, and MMT present in the Ag-MMT/CS colloid dressing exert synergistic effects and confer good biocompatibility, and it has the potential to become a next-generation clinical antibacterial dressing.

## Figures and Tables

**Table 1:** Orthogonal factors and level.

Level	Silver Nitrate Concentration A (mol/L)	Time B (h)	Temperature C (°C)	pH D
1	0.04	3	50	4.4
2	0.05	4	60	5.4
3	0.06	5	70	6.4

**Table 2:** Orthogonal test results.

Serial Number	Silver Nitrate Concentration (mol/L)	Time (h)	Temperature (°C)	pH	Silver Content (mg/g)
1	1	1	1	1	44.38
2	1	2	2	2	49.89
3	1	3	3	3	41.23
4	2	1	2	3	60.56
5	2	2	3	1	68.42
6	2	3	1	2	59.67
7	3	1	3	2	55.66
8	3	2	1	3	51.35
9	3	3	2	1	53.11
Mean value 1	45.167	53.533	51.800	55.303	
Mean value 2	62.883	56.553	54.520	55.037	
Mean value 3	53.373	51.337	55.103	51.047	
Range	17.716	5.216	3.303	4.256	

## Data Availability

The authors confirm that the data supporting the findings of this study are available within the article.
